# Modulation of cyanobacterial Photosystem I protein environment and spectral capacity in response to changes in electron flow pathways and photon flux

**DOI:** 10.1016/j.jbc.2025.110233

**Published:** 2025-05-14

**Authors:** Sharon L. Smolinski, Monika Tokmina-Lukaszewska, Junia M. Holland, Zhanjun Guo, Effie Kisgeropoulos, Brian Bothner, Paul W. King, Carolyn E. Lubner

**Affiliations:** 1 Biosciences Center, National Renewable Energy Lab, Golden, Colorado, USA; 2 Department of Chemistry and Biochemistry, Montana State University, Bozeman, Montana, USA

**Keywords:** photosynthesis, photosystem I, carotenoid, chlorophyll, cyanobacteria, oligomeric composition

## Abstract

Cyanobacterial photosystem I (PSI) can undergo modifications that adjust photosynthetic electron transport in response to fluctuations in environmental and cellular conditions. We recently reported that PSI isolated from *Synechocystis* sp. PCC 6803 (*S*. 6803) strains lacking a peripheral oxygen reduction reaction (ORR1) pathway demonstrated altered P_700_ photooxidation capacity, changes in spectral properties, and a higher proportion of monomers. These changes in PSI were augmented when cells were grown under higher photon flux, which creates a greater energy imbalance at PSI. We have shown that the modified PSI is functional in photochemical charge separation and ferredoxin reduction reactions. Thus, we hypothesized that monomerization of PSI was caused by changes in the environment of PsaL, which is known to be essential for stabilizing trimers. To test our hypothesis, we isolated PSI monomers and trimers from ORR1 and wild-type (WT) strains. The electron paramagnetic resonance (EPR) spectra of reduced PSI demonstrated the presence of intact F_A_ and F_B_ [4Fe-4S] clusters, consistent with measurements of functional charge separation and electron transport. Limited proteolysis followed by mass spectrometric analysis showed altered accessibility of PsaL in the ORRI PSI monomers compared to WT monomers, and included regions associated with chlorophyll and carotenoid binding, and in functional interactions with adjacent subunits. In addition, ORR1 PSI monomers had spectral changes compared to WT PSI due to differences in carotenoid compositions. Collectively, these findings reveal new insights into how microbes adjust PSI structure and photochemistry to mitigate photodamage in response to changes in electron utilization by downstream chemical reactions.

Cyanobacteria adjust photosynthetic electron flux in response to natural variations in photon flux through multiple mechanisms involving components of the photosynthetic electron transport (PET) chain. As core components of the PET chain, the Photosystem II (PSII) and Photosystem I (PSI) reaction centers work in tandem to convert photon energy into highly reducing electrons that connect with a network of downstream electron utilization pathways. The modulation of reaction center activity to varying environmental conditions allows for the maintenance of cellular function and leads to the generation of metabolites and chemical compounds relevant to many sustainable energy applications (1, 2, 3, 4, 5, 6). PSI, which interfaces PET with metabolic reduction-oxidation reactions, is a multi-subunit protein–pigment complex that has been shown to form monomers, dimers, trimers, and tetramers (7, 8, 9, 10) largely dependent upon the organism from which it has been isolated. In *Synechocystis* sp. PCC 6803, PSI occurs as both monomers and trimers, with the dominant form being the trimer. Trimers are comprised of 33 protein subunits, 285 light-harvesting chlorophyll *a* (chl*a*), and 72 carotenoid molecules, in addition to nine iron-sulfur clusters, six phylloquinones, and various lipids (11). The pigment network acts as an antenna to absorb and funnel light energy into the primary donor of the reaction center, P_700_, which then uses that energy to promote a transmembrane electron transfer event (12, 13) (Fig. 1). The importance of PSI photochemistry to photosynthetic electron transport in *Synechocystis* is evident from the two to five-fold greater abundance of PSI compared to PSII in the thylakoid membrane (14, 15).

**Figure 1:**
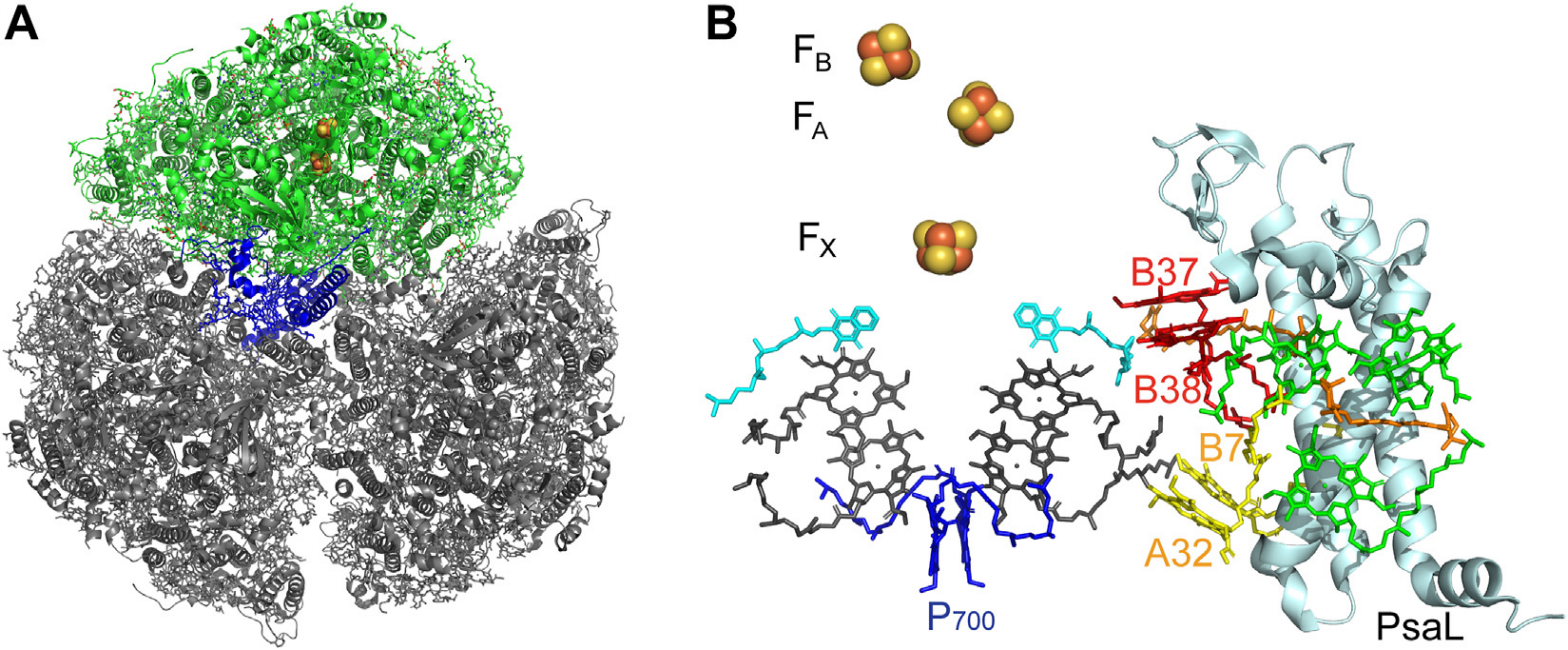
**Location of PsaL within the PSI complex**. *A*, PsaL (in *blue*) in relation to PSI trimeric structure as viewed from the stromal surface (one monomeric unit in *green*, two monomeric units in *grey*), based on PDB 5OY0, the structure of WT *Synechocystis* sp. PCC 6803 trimer (11). *B*, PsaL (*light cyan*) in relation to nearby chls (*green*), red chl pairs B37/B38 (*red*) and A32/B7 (*yellow*), and β-carotenes (*orange*). PsaL is also shown in relation to the electron transfer chain, P_700_ (*blue*), chlorophylls (*gray*), phylloquinones (*light blue*), and iron-sulfur clusters (*yellow* and *orange spheres*). Stromal side is at the *top*. Based on PDB 5OY0, the structure of WT *Synechocystis* sp. PCC 6803 trimer (11), and red chls identified in (13, 22). Features depicted in B are specified in Table S1.

The causes for and mechanisms by which cells adjust oligomeric structure or modulate photochemistry are not well understood. In particular, the functionality and photochemistry of PSI monomers warrants further investigation to understand if there is an energetic advantage of this oligomeric form. To begin to address these questions, we utilized a strain of *S.* 6803 deficient in the oxygen reduction reaction (ORR) catalyzed by flavodiiron 1 (ORR1), to curtail electron consumption downstream of PSI. We previously found that the loss of the ORR1 pathway resulted in pronounced changes in PSI oligomeric, spectral, and photochemical properties (15). For example, PSI from the ORR1 strain displayed decreased contributions from long-wavelength chls (known as “red” chls), attenuated photooxidative capacity of P_700_, and a greater proportion of monomeric PSI than was observed for PSI from the WT strain. When ORR1 was cultured under increased light, the greater imbalance between energy input and demand amplified all these observed changes. Under the increased light condition, ORR1 PSI retained the ability to conduct photochemistry in both whole cells and isolated reaction centers but showed clear differences in kinetic rates and light saturation profiles compared to WT. These differences, however, did not impair the ability of ORR1 PSI to donate electrons to the native redox partner ferredoxin. Intriguingly, PSI monomerization increased when ORR1 was cultured under moderate illumination (1.4-fold) or under increased fluctuating light conditions (5-fold) (15). The changes in PSI oligomeric composition and photochemistry observed in the ORR1 strain support a rebalance in cellular energy, resulting in only moderate growth effects (15, 16, 17). However, the physical properties of PSI centers that are modified in ORR1 strains to accomplish this remain to be established.

While it is well known that the PsaL subunit plays an important role in stabilizing the trimeric form of PSI (18), this subunit was retained in both the trimers and monomers from ORR1. Other studies have established that deletion or modification (for example, through addition of a single His residue) of PsaL induces absolute or predominant monomerization of PSI (11, 18, 19). Furthermore, effects on the amount and spectral properties of pigments, including for chls, β-carotene, zeaxanthin, and echinenone, as well as interactions with other subunits in the trimerization region have also been reported in the studies which directly manipulated PsaL (19, 20, 21, 22). This subunit is adjacent to multiple red chls that function to extend the capacity for light absorption further red than the P_700_ primary donor (12, 23), and are thought to increase the overall efficiency of photochemical trapping (22). Among the red chls reported for *S.* 6803 PSI, two pairs are located near the PsaL subunit (shown in Fig. 1): B37 and B38 are assigned to PsaB, and A32 and B7 are assigned to PsaA and PsaB, respectively (13, 22). The latter pair is hypothesized to lose their long-wavelength character in PSI monomers when PsaL is absent (22), suggesting a direct functional role of PsaL in the adjustment of PSI spectral and photochemical properties. Thus, the modulation of PSI photochemical properties, through effects in the PsaL subunit, may provide a powerful control valve for cells to integrate energy input (*i.e.* light availability) with output (*i.e.* downstream electron utilization pathways). Understanding the role of PsaL, and other factors that affect this control of PSI properties, may benefit future metabolic engineering approaches.

Here we investigated the physical properties of PSI centers for ORR1 to determine whether PsaL is directly involved in the modification of PSI function by analysis of PSI isolated from cells cultured under moderate (growth light, GL) or increased and fluctuating light (FL) conditions. Using a combination of limited proteolysis and mass spectrometry, changes in the accessibility and dynamics of PsaL were mapped onto the structural models. The results showed significant alterations in the proteolytic cleavage site accessibility of PsaL in PSI from ORR1 strains compared to WT, indicating differences in the PsaL protein environment. Importantly, differences were observed in regions associated with or proximal to pigment-binding sites. The analysis also revealed decreased interactions with select subunits adjacent to PsaL in ORR1. Concurrent with these observations, carotenoid abundance and composition of PSI monomers and trimers were found to be altered in ORR1 while the integrity of the electron transfer chain within PSI was intact as probed by EPR. These adjustments in the protein environment of PsaL can serve as a physical mechanism to support the previously observed changes in PSI photochemical properties (15), collectively facilitating the modulation of energy flux at PSI. The observations of the functional plasticity of PSI explain how subtle variations in PsaL impact the spectral capacity and photochemistry of PSI to enable cells to meet energetic demands under dynamic growth conditions. Furthermore, this plasticity is observed in naturally occurring PSI monomers, a form of PSI that is currently underexplored. This work adds new insights to the understanding of how the complex PSI molecular machine functions to balance energy flux.

## Results

### Integrity of the terminal electron transfer pathway iron-sulfur clusters in ORR1 PSI monomers and trimers

**Figure 2:**
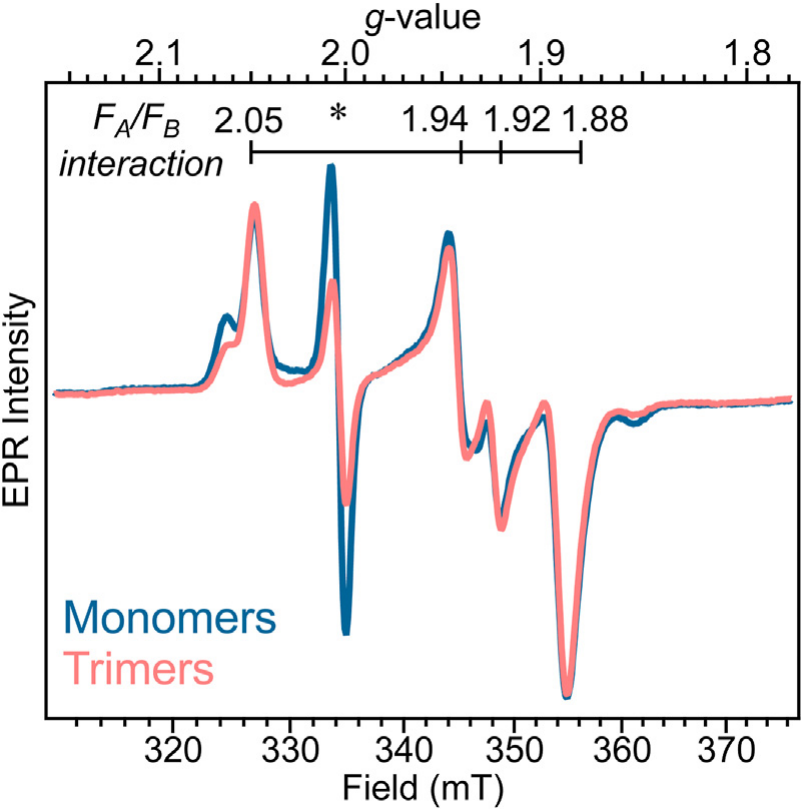
**CW EPR spectra of PSI monomers and trimers.** EPR analysis of PSI monomers (*teal*) or trimers (*pink*) isolated from the ORR1 strain grown under FL conditions. Samples were frozen under room light conditions after chemical reduction using 5 mM sodium dithionite. The characteristic interaction spectrum of reduced F_A_/F_B_ in PSI complexes is observed with features at *g* = 2.05, 1.94, 1.92, and 1.88. An asterisk denotes the P_700_ radical. Wing features at *g* ∼1.85 and 2.07 indicate a small (5–10%) contribution to the signal from either reduced F_A_ or F_B_ alone. Data collected at 15 K and 1 mW of power. Sample chlorophyll concentration at 1.1 mg/ml (trimers) or 2.02 mg/ml (monomers).

Our previous study of PSI isolated from the ORR1 strain demonstrated that both monomeric and trimeric forms were functional and able to carry out photochemistry, albeit with decreased P_700_ photooxidative capacity compared to WT (15). To further confirm the functionality of PSI monomers and trimers from ORR1, EPR spectroscopy was performed to examine the integrity of the terminal electron transfer acceptors, the F_A_ and F_B_ iron-sulfur clusters (Fig. 1*B* and Table S1). When both these clusters are reduced, either by promotion of multiple electrons through P_700_ using continuous illumination or through chemical reduction by incubation of PSI with sodium dithionite at pH 10, a characteristic “interaction spectrum” (*g* = 2.05 1.94 1.92 1.88) is observed by EPR (24). This signature is the result of spin-spin coupling between the two nearby clusters, which are paramagnetic when reduced, and is distinct from the EPR signals originating from just reduced F_A_ or reduced F_B_. The EPR spectra of chemically reduced ORR1 PSI monomer and trimer samples collected at 15 K (Fig. 2) display the F_A_/F_B_ interaction spectrum as reported for WT (24), confirming that both clusters are intact and functional in ORR1.

### PsaL accessibility is altered in ORR1 monomers

**Figure 3:**
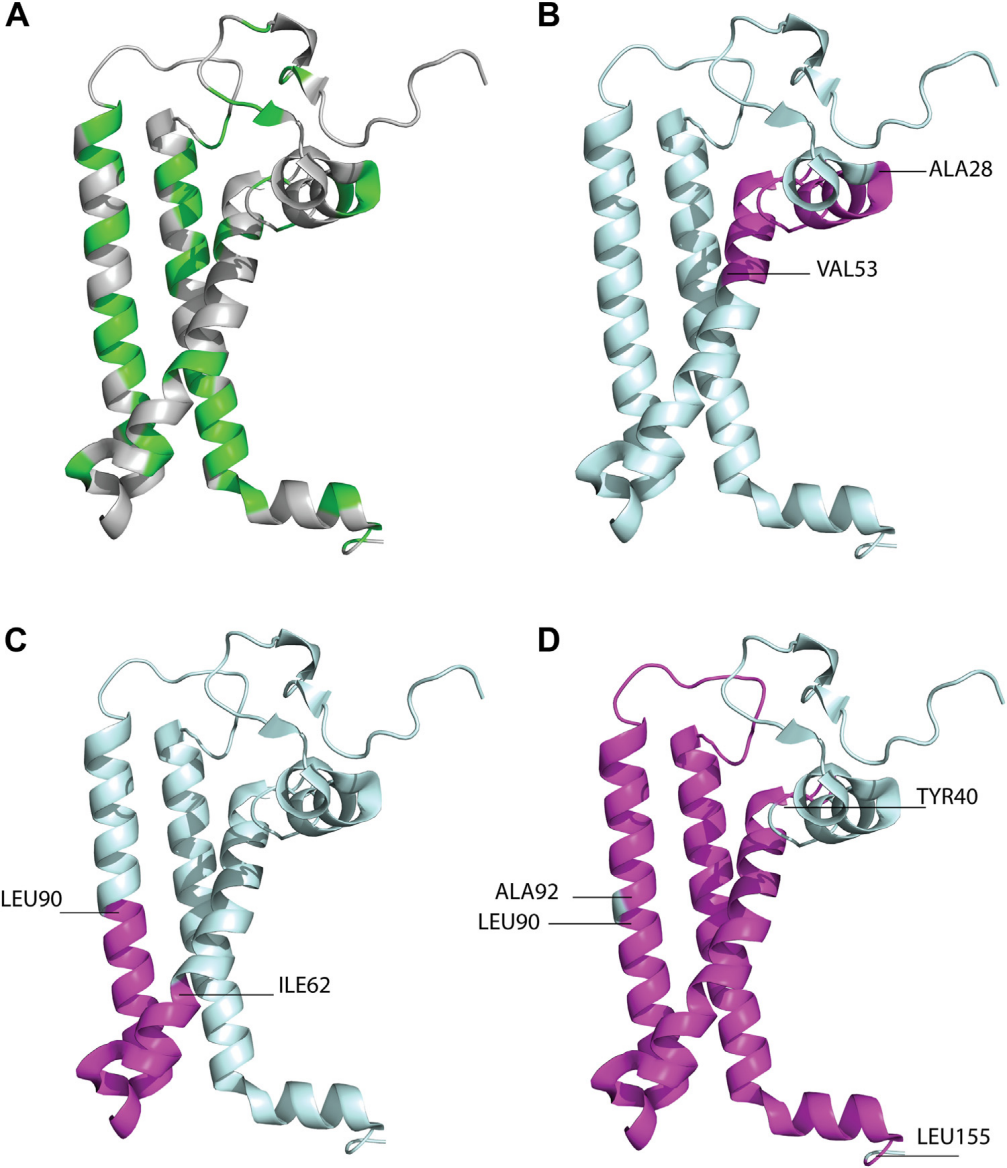
**Regions of PsaL detected by mass spectrometric analysis following limited proteolysis**. Observed peptides are mapped onto the structure of *S*. 6803 WT PsaL from PDB 5OY0 (11). *A*, potential pepsin cleavage sites shown in *green* (see list in Table S2). PsaL regions detected following proteolysis are in *magenta* (panels *B* and *C*). *B*, WT PSI trimers (ALA28–VAL53), (*C*) WT PSI monomers (ILE62–LEU90), and (*D*) ORR1 PSI monomers (TYR40–LEU90 and ALA92–LEU155) from corresponding strains grown in FL. PsaL is oriented with the stromal side on *top*, as viewed from PsaA and PsaB.

Since the basic functionality of PSI is retained in PSI monomers and trimers from ORR1 cultured under GL and FL growth conditions, including the presence of the PsaL subunit (15), we hypothesized that the shift in oligomeric state might be associated with modulation in the protein environment surrounding PsaL. Subunits adjacent to PsaL include PsaA, PsaB, PsaD, PsaI and PsaM subunits. To examine this possibility, we employed limited proteolysis (25, 26) followed by mass spectrometry to determine the relative accessibility and dynamics of PsaL in PSI monomers isolated from cells grown under FL conditions. Pepsin was used for proteolysis owing to the high number of cleavage sites, 61, in PsaL (Table S2) compared to only six for trypsin (27), which results in higher sequence coverage and more data points for more robust comparisons between PSI samples. The datasets of detected peptides (Table 1) were combined for each strain and condition (Table 2) and subsequently mapped onto the crystal structure of WT *S.* 6803 PSI from PDB 5OY0 (11) (Fig. 3). This mapping of detected peptides onto the WT structural model allows for the comparative visualization of the accessibility of PsaL, including how this may correspond to associated pigments.

**Table 1:** Peptides detected by mass spectrometric analysis of PsaL in isolated WT trimers and monomers, and ORR1 monomers

Strain and growth condition	Number of peptides	Peptide mass	N and C terminal ends of PsaL peptides (± mass error, Da)^a^	Positions of nearest pepsin cleavage sites to detected peptides ends	Number of pepsin cleavage sites near or within detected peptide regions
WT FL Trimer	1	2890.5	ALA 28–VAL 53 (−0.08)	28, 50	7
WT FL Monomer	1	3113.6	ILE 62–LEU 90 (+0.07)	61, 90	17
ORR1 FL Monomer	4	3884.6	TYR 40–SER 74 (−0.02)	40, 75	11
		3113.6	ILE 62–LEU 90 (+0.07)	61, 90	17
		6572.6	ALA 92–LEU 155 (+0.03)	90, 154, 156	31
		1969.0	PHE 107–ALA 125 (−0.03)	104, 126	8

a PsaL peptide fragments obtained from pepsin digestion are numbered to indicate the pepsin cleavage site. The number in parentheses are the mass error in Da.

**Table 2:** Peptides detected by mass spectrometric analysis of PsaL from isolated WT and ORR1 PSI trimers or monomers following proteolytic digestion with pepsin, and the percentage of peptides detected of total peptides for the subunit

Strain and growth condition	Aggregated peptides detected in PsaL	Detected peptides as % of subunit
WT Trimer	ALA 28–VAL 53	17%
WT Monomer	ILE 62–LEU 90	19%
ORR1 Monomer	TYR 40–LEU 90, ALA 92–LEU 155	73%

We observed differences in the accessibility of PsaL in WT PSI monomers compared to WT trimers (Tables 1 and 2, Fig. 3). This comparison between WT oligomeric forms can serve as a benchmark for what to expect for changes due primarily to monomerization. Our analyses detected 17% of the PsaL sequence from the WT PSI trimer sample compared to 19% from the WT PSI monomer, with the detected peptides located in different regions of the subunit. In the WT PSI trimer, ALA28 to VAL53 was detected, whereas ILE62 to LEU90 was observed in the WT PSI monomer. The ILE62 to LEU90 region, which is at the trimerization interface of PsaL, is more buried and blocked from proteolysis in the trimers by interactions between monomeric units but becomes accessible in WT monomers (Fig. 3*B* *vs* Fig. 3, *C* and *D*). On the other hand, ALA28 to VAL53 is at the protein-protein interface between monomers of a trimer. These data indicate that in solution, PsaL has distinctly different conformations when comparing the trimer and monomer, and the identified regions are important for the specific oligomeric state.

The accessibility of PsaL to proteolysis by pepsin was even greater in ORR1 PSI monomers compared to WT PSI monomers (Fig. 3*C* *vs* Fig. 3*D*, respectively) isolated from cells cultured under the same conditions. In the ORR1 PSI monomers, the accessible regions comprise a larger proportion of PsaL and potential pepsin cleavage sites (Tables 1 and 2). In the ORR1 PSI monomer, 73% of the PsaL sequence was detected compared with 19% in the WT PSI monomer sample. The detected regions in ORR1 monomer PsaL were TYR40 to LEU90 and ALA92 to LEU155, compared to the overall shorter region of ILE62 to LEU90 in WT PSI monomers. The regions of increased accessibility in ORR1 PSI monomers included the trimer interface region and across much of the transmembrane domains of PsaL. The enhanced accessibility of PsaL in ORR1 is in addition to the effects arising from monomerization alone. These findings indicate that multiple sites in PsaL, which were previously inaccessible to proteolysis under the conditions tested, became available in PSI from ORR1, and indicate dissimilarities in the protein environments of PsaL between ORR1 and WT.

**Figure 4:**
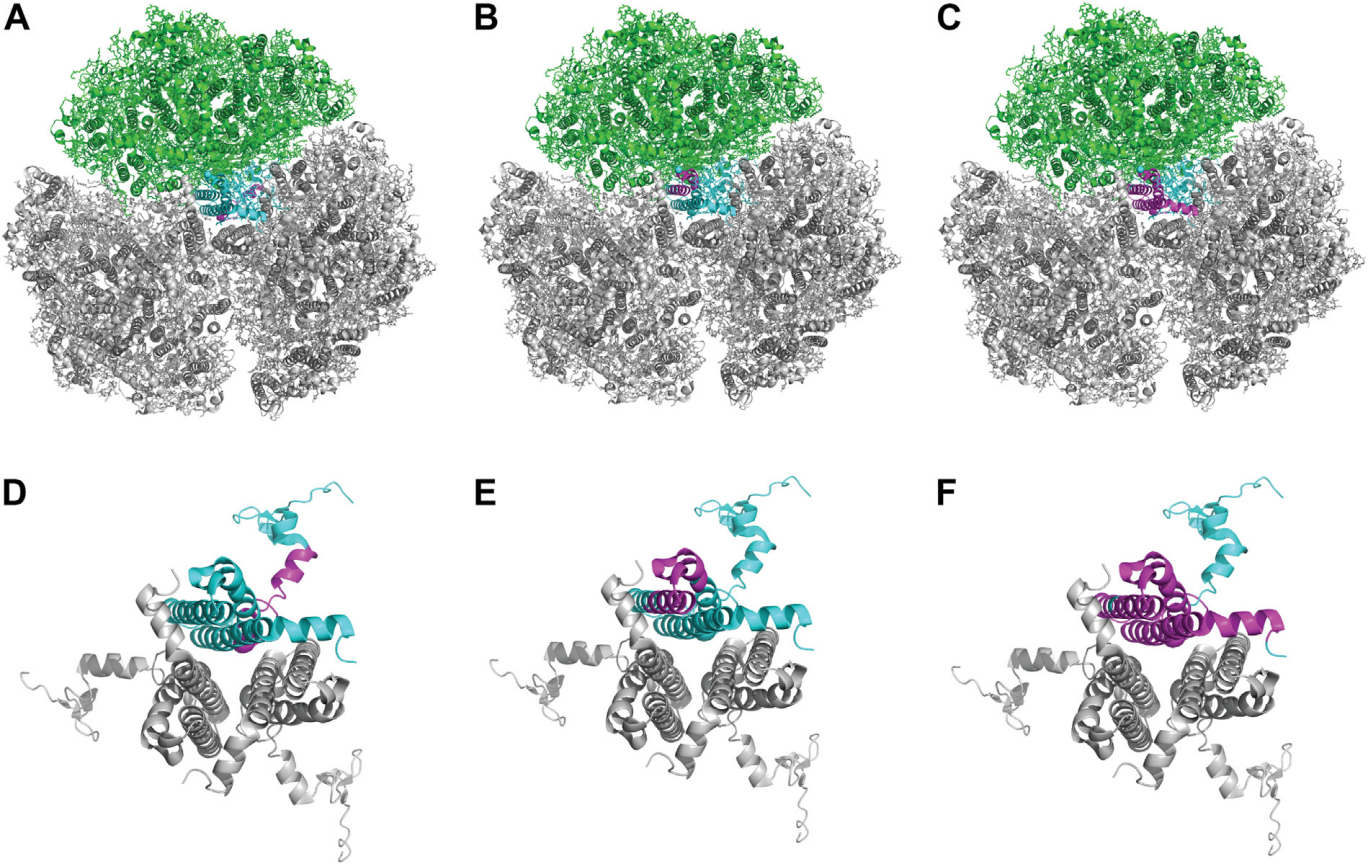
**Regions of PsaL detected following limited proteolysis of PSI monomers and trimers.** Data are mapped onto the trimeric structure of *S*. 6803 WT PSI from PDB 5OY0 (11). for data collected from PSI trimers and PSI monomer samples. Accessible residues observed for PsaL from (*A*) WT PSI trimer, (*B*) WT PSI monomer, and (*C*) ORR1 PSI monomer cultured under FL. PsaL is in *cyan* and detected peptides in *magenta*. For clarity a monomeric unit is shown in *green*. The figures are rendered to depict the physical relation of the monomer with respect to the trimer (*gray*). Magnified PsaL subunits; (*D*) WT PSI trimer, (*E*) WT PSI monomer, and (*F*) ORR1 PSI monomer. A single PsaL subunit representing the PSI monomeric structure is color-coded in *cyan* with the detected peptides in *magenta*. The two additional PsaL in *gray* correspond to neighboring monomeric units in trimeric form.

Regions of increased accessibility to proteolysis in PsaL in the ORR1 PSI monomer, compared to WT, correspond to areas where monomeric units interact in the trimeric structure and include most of the C-terminal end of PsaL, up to LEU155 (Fig. 4). This increased accessibility of the C-terminal region in PsaL of the ORR1 PSI monomer indicates changes in the protein environment that likely have consequences for trimer formation and/or stability and may contribute to the increased proportion of monomeric PSI we previously reported for this strain and condition.

**Figure 5:**
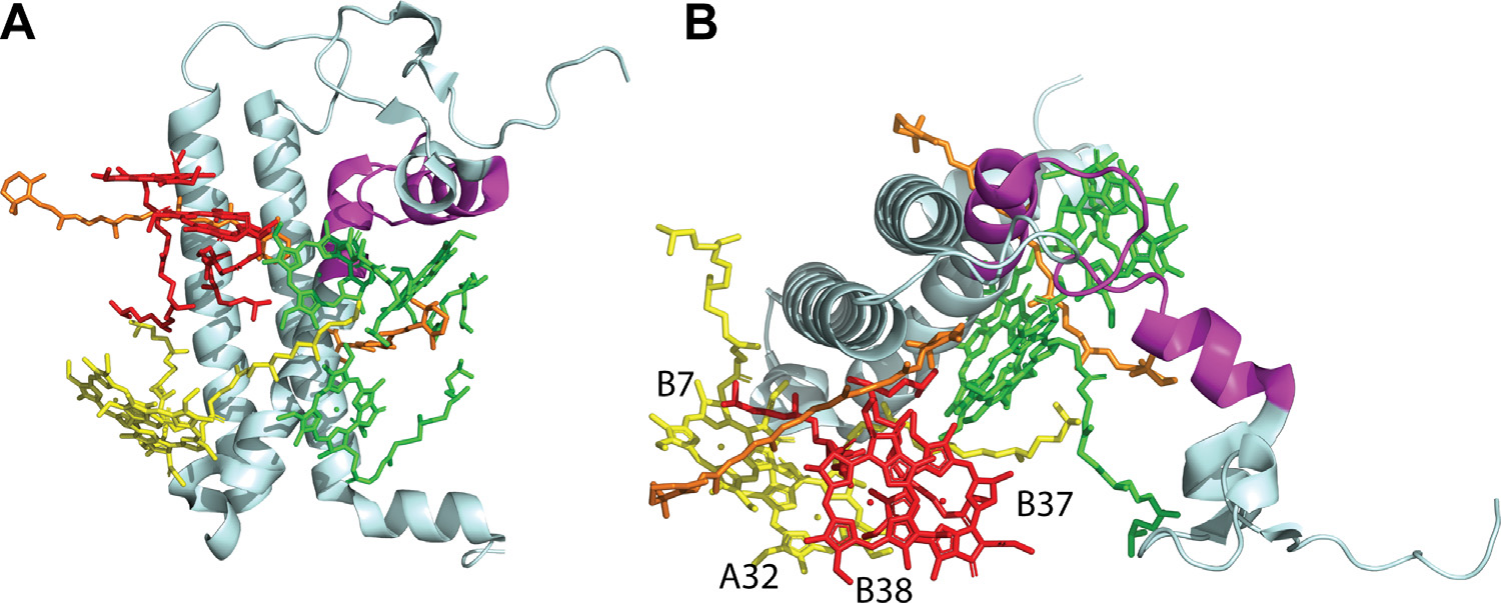
**Maps of the PsaL accessibility regions to the chlorophyll and β-carotene binding regions of PsaL in the *Synechocystis* PSI trimer structure**. PSI pigments from the *S*. 6803 PSI trimer structure PDB 5OY0 (11) are shown overlaying the accessibility regions of PsaL (*magenta*) in ORR1 PSI trimers. *A*, stromal side on top and (*B*) stromal side looking toward the lumenal side. PsaL in *light cyan* with detected regions of PsaL in *magenta*, three chls (*green*), two β-carotenes (*orange*), red chl pairs B37/B38 (*red*), and A32/B7 (*yellow*).

Multiple pigments are associated with or near PsaL based on the structure of *S*. 6803 WT trimeric PSI (Fig. 5), including three chls and two β-carotenes (11) (shown in green and orange, respectively). Red chl pairs B37/B38 (shown in red) and A32/B7 (shown in yellow) are adjacent to PsaL, with the closest points between the red chls and PsaL ranging between 4 and 6 Å based on the reported *S*. 6803 WT trimeric PSI structure (11) (Fig. S1). Our findings show increased accessibility in ORR1 PSI monomers for all regions associated with pigments in the WT trimer structure, including regions associated with chls, red chls, and carotenoids (Fig. 5). Further, red chl pair A32 and B7 (shown in yellow), assigned to PsaA and PsaB, are adjacent to the PsaL subunit and found within the regions of increased accessibility in the WT monomer, consistent with decreased red chl emissive properties in PSI monomers generated using a deletion of *psaL* (22). These findings of increased accessibility indicate a dissimilar protein environment of PsaL in ORR1 PSI monomers, which may have consequences for the associations with pigments, but which have not yet been assessed for this strain and condition.

### Interactions between PsaL and adjacent subunits are altered in ORR1

Given the evidence of an altered PsaL protein environment in ORR1 PSI monomers, we assessed additional mass spectrometry data on both PSI trimers and monomers for changes in interactions between PsaL and adjacent subunits. This analysis focused on amino acid residues within 5 Å of PsaL, here referred to as the interaction sphere, based on the existing crystal structure of WT PSI trimer (5OY0) (11). PSI monomers and trimers isolated from cells grown in FL conditions were treated with proteolysis with pepsin for 10 min and analyzed to identify undetected residues within the interaction sphere and compared across different strains and oligomeric forms. The 10-min pepsin digestion patterns were analyzed to investigate the protein-protein interaction network within the PSI complex in the context of the oligomeric state. Our analysis was divided into three parts (see Supporting information). First, the interaction reference list was generated for each of the three PsaL subunits in trimeric PSI. The reference list, constituting the “interaction sphere”, contained amino acid residues and cofactors from the nearest environment of PsaL. The nearest environment is the collection of interactions within 5 Å distance, based on the crystal structure of the PSI trimer (PDB 5OY0) from *S. 6803* (11). The interaction sphere of PsaL subunit was made of a maximum of 14 aa from PsaA, 29 aa from PsaB, 16 aa from PsaD, 27 aa from PsaI, and 13 aa from PsaM which represents about 2%, 3.5%, 10.5%, 65%, and 38% of the entire subunit sequence, respectively. In the next step, the PsaL pepsin digestion patterns in each sample were investigated. Differences in the digestion pattern, which is the absence of peptide fragment(s) (*i.e.* undetected peptides) were interpreted as being caused by a change in the accessibility of that cleavage site and provide evidence of altered regions of interaction between the PsaL subunit and adjacent subunits and cofactors. Only the PsaL protein regions which remained undetected and were localized to the corresponding interaction spheres were selected for further comparisons. In the final step, undetected regions of the PsaL interaction sphere were investigated using pairwise comparisons between strains (WT and ORR1) and PSI oligomeric forms (trimers and monomers) from cells cultured under FL conditions to reveal residues that were detected in one strain and form but not in another. These comparisons provide insights into changes in interactions between PsaL and neighboring subunits, for: WT monomers compared to WT trimers, ORR1 monomers compared to WT monomers, ORR1 monomers compared to ORR1 trimers, and ORR1 trimers compared to WT trimers. For spatial context, the differences for each comparison were overlaid on the PSI trimer structure (PDB 5OY0) from *S. 6803* (11). Since the interaction network of PsaL was different between protomers (based on 5OY0), the results were overlaid on the three protomers, regardless of the sample’s actual oligomeric state. The differences in this interaction sphere are useful to probe how the function of energy capture, conversion, and transfer in PSI may be affected by structural changes.

**Figure 6:**
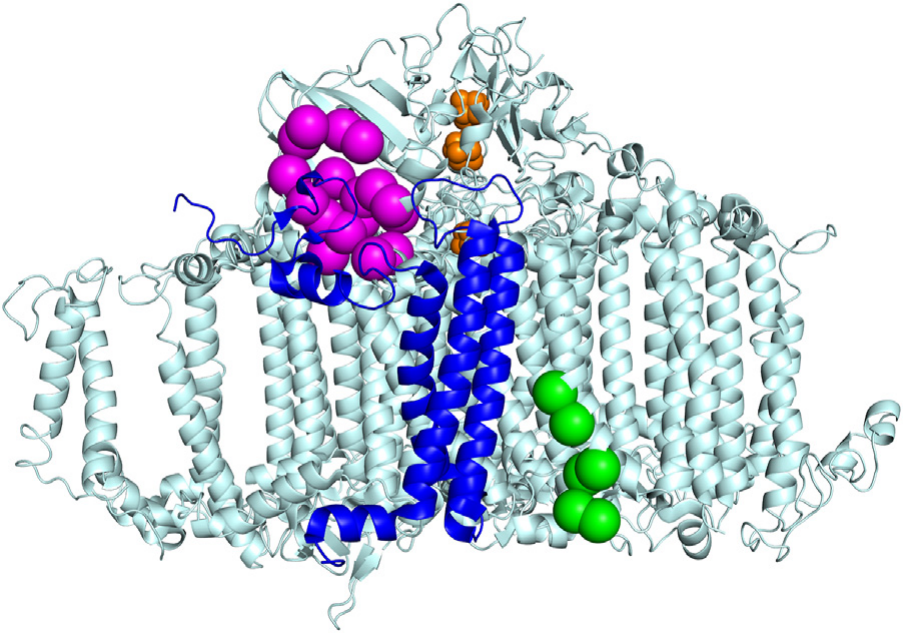
**The detection of residues within 5 Å of PsaL in ORR1 PSI monomer compared to WT monomer.** Residues that were not detected in ORR1 PSI monomer but are detected in WT monomer are shown in *magenta*. Residues detected in ORR1 PSI monomer but not detected in WT monomer are shown in *green*. Data are based on mass spectrometric analysis following proteolytic digestion and mapped onto a monomeric unit based on the structure of *S*. 6803 WT PsaL from PDB 5OY0 (11). PsaL is shown in *blue* and iron-sulfur clusters in *orange*. PSI is shown with the stromal side on *top*.

A comparison of ORR1 PSI monomers to WT PSI monomers identified 17 residues (per monomer) in the PsaL interaction sphere which were detected in WT monomers but not in ORR1 monomers, shown in magenta in Figure 6 (PsaL residues listed in Table S3), involving residues in PsaA, PsaB, and PsaD on the stromal side of PSI. Additionally, six residues were identified in the ORR1 PSI monomer that were not detected in WT monomer, shown in green (Fig. 6), which were located in PsaB and PsaI toward the membrane embedded and lumenal portion of PSI that faces PsaL. These changes in the accessibility of residues around PsaL suggest differences in interactions and proximity between PsaL and adjacent subunits that are specific to ORR1 monomers and not observed in WT.

**Figure 7:**
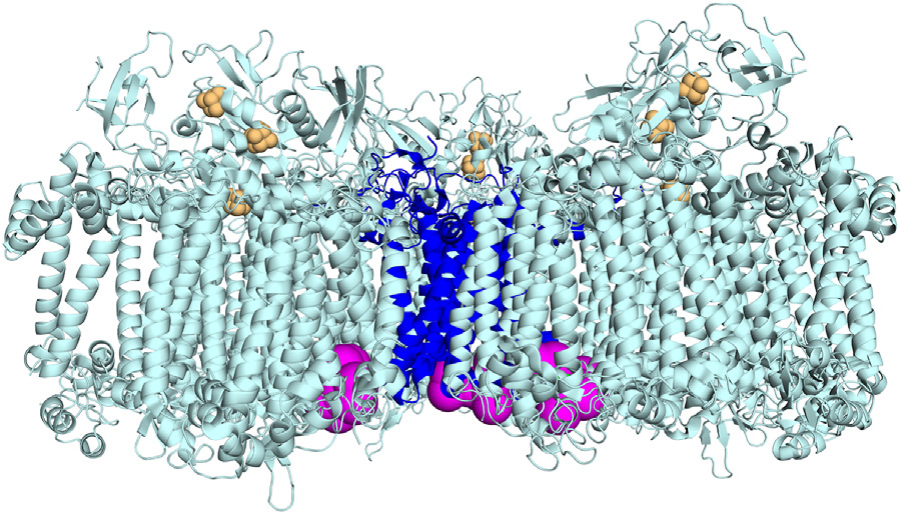
**The detection of residues within 5 Å of PsaL in ORR1 PSI trimer compared to WT trimer.** Residues within the PsaL interaction sphere that were not detected in ORR1 PSI trimer compared to WT trimer are shown in *magenta*, located on the lumenal side. Data are based on mass spectrometric analysis following proteolytic digestion, and mapped onto the trimeric structure of *S*. 6803 WT PsaL from PDB 5OY0 (11). PsaL in *blue* and iron-sulfur clusters in *yellow*. PSI is shown with the stromal side on *top*.

A comparison of ORR1 PSI trimers to WT PSI trimers identified a total of 27 residues, nine residues per monomeric unit, in the PsaL interaction sphere which were detected in WT trimer but not in ORR1 trimer, shown in magenta in Figure 7 (PSI subunit residues listed in Table S4). All of the residues not detected for the ORR1 trimer in this pairwise comparison were located on the lumenal side of PSI, in PsaA and PsaB. These findings show that there are differences in the accessibility of residues within the PsaL interaction sphere for ORR1 monomers and trimers compared to WT.

**Figure 8:**
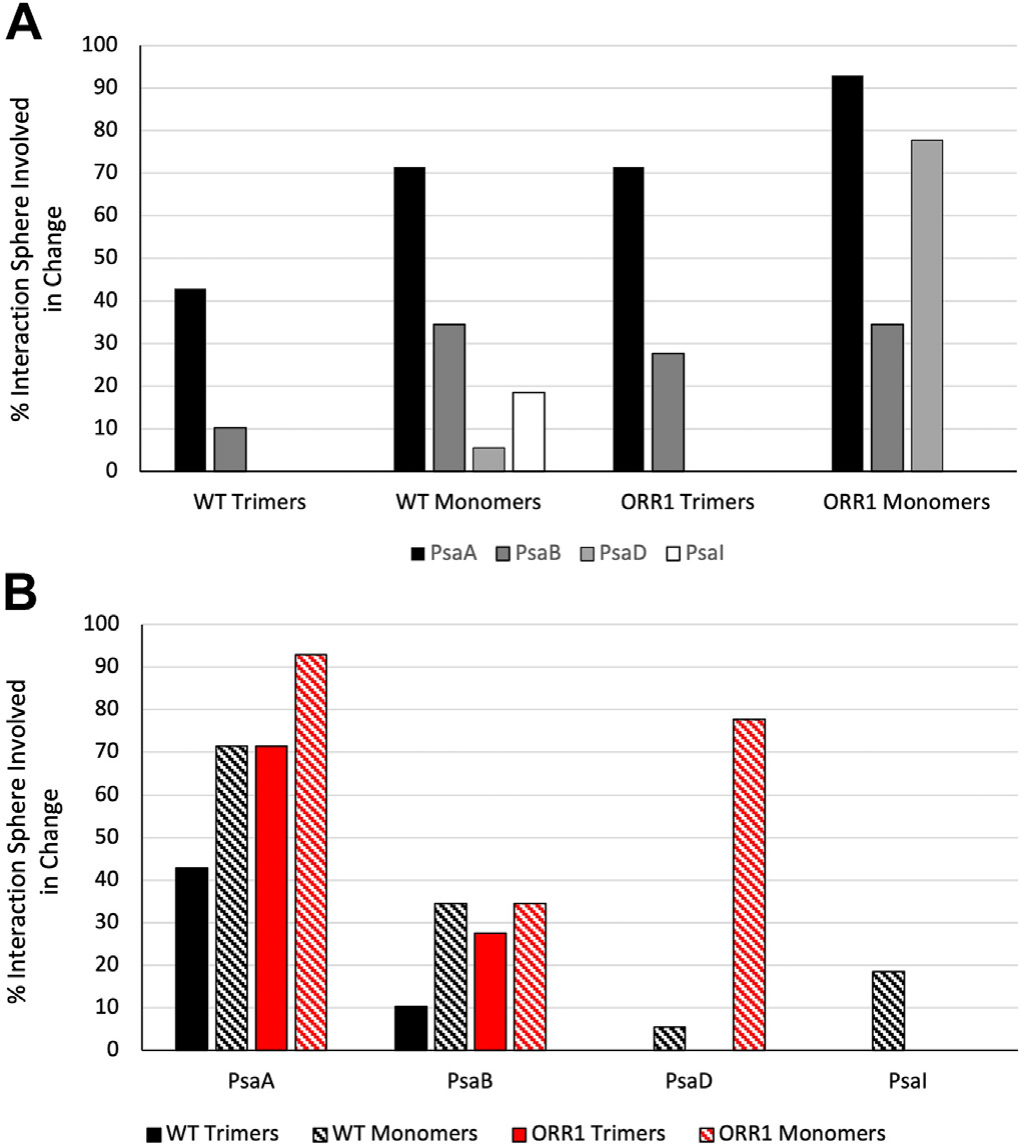
**The percentage of undetected residues in the PsaL interaction sphere shown as grouped comparisons by *A*) strains and PSI oligomeric form, and *B*) PSI subunit.** WT and ORR1 PSI trimers and monomers were isolated from cells grown under FL conditions, and the PsaL interaction sphere was mapped based on mass spectrometric analysis following proteolytic digestion and compared to the structure of *S*. 6803 WT PsaL from PDB 5OY0 (11).

Further, a comparison of WT PSI monomers with WT PSI trimers identified 15 residues detected in the trimeric form but not the monomeric form, primarily located on the lumenal side (Fig. S2, PSI residues listed in Table S5). However, the comparison of ORR1 monomers with WT monomers revealed differences on the stromal side of PSI. Comparing the residues detected in all strains and conditions (Tables S3, S4, and S5), the ORRI monomers had the greatest number of undetected residues. Additionally, there is variation in the subunits involved (Fig. 8) as well as the locations of the residues (stromal vs. lumenal). These variations in the total number and location of residues can have different impacts on energy transfer within and from PSI, and potentially for mechanisms related to adjusting oligomeric form.

### Characterization of carotenoid levels in PSI monomers and trimers

**Figure 9:**
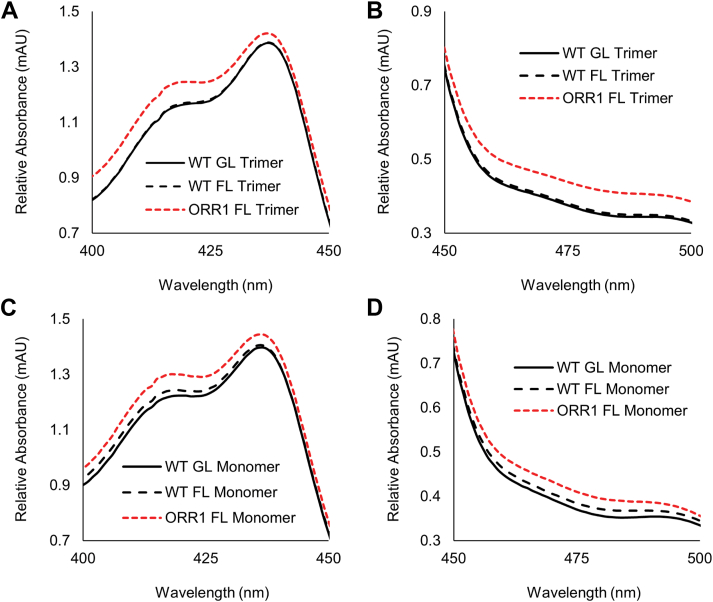
**UV-VIS absorption spectra of PSI isolated from WT and ORR1 cells grown under GL and FL.** PSI trimers over (*A*) 400 to 450 nm, and (*B*) 450 to 500 nm. PSI monomers (also containing PSII) over (*C*) 400 to 450 nm, and (*D*) 450 to 500 nm. ORR1 PSI trimers, and ORR1 PSI monomers (with PSII) show increased absorbance from 400 to 440 nm and 460 nm to 500 nm. Data were normalized to the peak for chl *a*, at 676 nm to 678 nm. Data are the average of biological triplicates.

To assess overall carotenoid abundance in PSI under conditions of mismatched electron demand and light availability, UV-VIS spectra of samples containing isolated PSI trimers and monomers were collected from 400 to 500 nm, the region for absorbance by carotenoids. The isolated PSI monomer samples additionally contained PSII, which cannot be differentiated in the analysis of PSI monomeric samples. Compared to WT, an overall increased absorbance is evident for samples of ORR1 PSI trimers and monomers from FL, particularly from 400 to 440 nm and 460 to 500 nm (Fig. 9). This overall increased absorbance is greater for ORR1 under FL conditions compared to GL conditions (Fig. S3). Multiple carotenoids absorb similar wavelengths across this region, signifying that there are broad differences in overall carotenoid abundance for ORR1 PSI trimers and samples containing both PSI monomers and PSII, when cultured under both light regimes but to a greater extent under FL conditions. Furthermore, carotenoids are increased in samples containing WT PSI monomers under FL compared to WT in GL, from 400 to 435 nm and 460 to 500 nm, indicating that an increased amount of carotenoids is a response to increased light in WT. However, the increase in carotenoid abundance is greater in samples of ORR1 PSI monomers compared to WT PSI monomers under FL conditions. In contrast, WT PSI trimers grown under FL conditions do not show a difference compared to WT trimers grown under GL, while WT PSI monomers and PSII grown under FL show an increase compared to WT GL but to a much lesser extent compared to ORR1.

### Analytical quantification of carotenoids in PSI monomers and trimers

**Figure 10:**
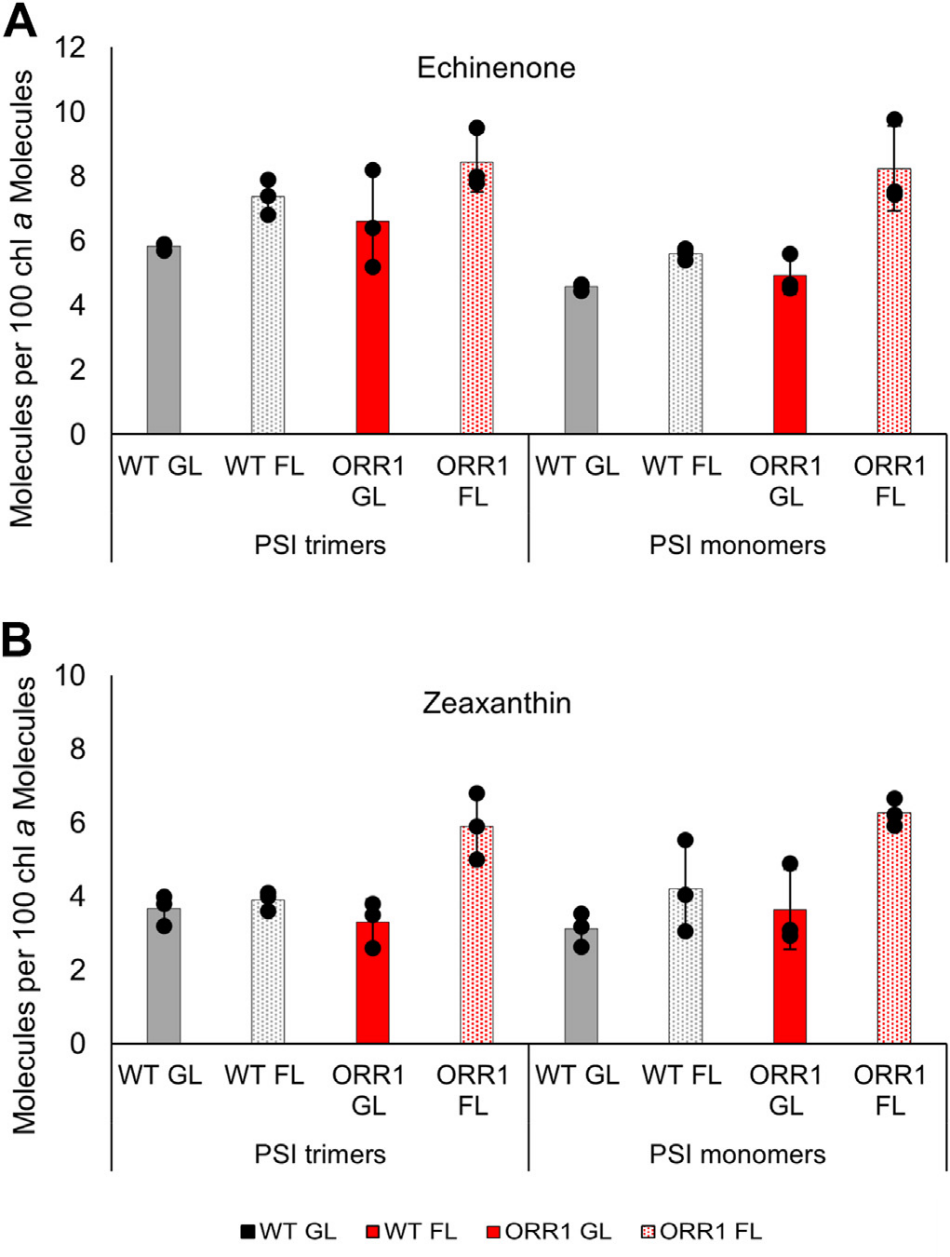
**Analytical measurements of carotenoid levels in PSI samples.** The abundance of (*A*) echinenone and (*B*) zeaxanthin in pigments extracted from samples of isolated PSI trimers and monomers from WT (*black*) and ORR1 (*red*) cells grown under GL (*solid*) and FL (*pattern*). Data are based on absorbance at 460 nm for echinenone and 450 nm for zeaxanthin and quantified as the number of molecules per 100 chl*a* molecules. Data are the average of biological triplicates, with the exception of ORR1 FL trimer for echinenone and zeaxanthin which have two biological replicates to exclude outlier values, with error bars showing standard deviation.

To better understand the carotenoid composition in ORR1 PSI trimers and monomers, the pigments were extracted, analyzed by HPLC, and quantified at 460 nm for echinenone, 450 nm for zeaxanthin, and 454 nm for β-carotene (Figs. 10, S4, and Table S6). Echinenone content in WT PSI increased under FL conditions, but to a greater extent in combination with the loss of ORR1. In WT PSI, echinenone levels were increased 26% and 22% for trimers and monomers under FL compared to GL. Echinenone content was even greater in ORR1 PSI under FL, with levels 45% and 78% greater for ORR1 PSI trimers and monomers compared to WT GL. Zeaxanthin content was increased in ORR1 PSI compared to WT GL. Changes in samples containing PSI monomers may also potentially reflect changes in carotenoid content of PSII. These results demonstrate that there are modifications of both echinenone and zeaxanthin abundance under a combined increased photon availability and attenuated electron utilization in ORR1. In addition, the overall abundance of β-carotene is increased in samples containing ORR1 PSI monomers under GL relative to WT. Overall, these data demonstrate that there are adjustments in the abundance of β-carotene in PSI under different light conditions in WT, and this response is altered upon the loss of the ORR1 pathway.

## Discussion

The findings in this study demonstrate changes in the protein environment of PsaL in PSI monomers and trimers from an ORR-deficient strain, which have implications for energy transfer internal and external to PSI. These results support our hypothesis that the photochemical and spectral changes in PSI monomers and trimers from an ORR-deficient strain (15) result from changes in the protein environment of PsaL. These modulations in PSI properties enable the re-tuning of energy transfer and dissipation pathways within PSI when there are variations between energy inputs (*e.g.* light availability) and outputs (*e.g.* utilization of electron flow) at PSI. Moreover, our findings show the functionality of the ORR1 PSI monomer is maintained, as evidenced by intact iron-sulfur clusters that facilitate the photochemical activity previously observed for these PSI particles (15). Collectively, this work supports that diversity in oligomeric form and spectral and photooxidative properties (15) are functional ways to enable responsive adjustments in energy management at PSI.

Associations with red chls and other pigments are likely to be impacted by the differences in the protein environment in and around PsaL in ORR1 PSI. For example, greater accessibility of the subunits found within the monomer interface could lead to alterations in the hydrophobicity and dielectric fields around co-located pigments. Additionally, structural deviations either within the affected subunits or to the quaternary structure of PSI may act to modulate the orientation and/or binding of pigments, and hence their spectral and energetic properties and contributions. ORR1 PSI trimers and monomers have been shown to exhibit decreased red chl emissive properties and attenuated P_700_ photooxidation as well as diminished electron transfer to flavodoxin (15), which is in agreement with modifications to the microenvironments around red chls and carotenoids found near PsaL. Specifically, the A32/B7 pair of red chls has been assigned as the fastest pathway of effective energy transfer from the antenna to P_700_, while other red chls may harbor the lowest-energy state (22). Among those, the B37/B38 pair is also found near the regions of PsaL (based on the WT PSI structure) whose accessibility is affected in ORR1, implying that energy transfer between the bulk antenna and the P_700_ primary donor is likely perturbed at this node. The pronounced changes in accessibility within this region of PsaL correlate with the modulation of PSI photochemistry and reduced forward electron transfer seen in the ORR1 PSI monomers.

Interactions with protein partners such as ferredoxin (Fd) or PBS may be impacted in ORR1 PSI monomers, given the changes in the accessibility of neighboring subunit residues on the stromal side of ORR1, which suggest shifts in the relative proximity of subunits. Based on the interaction sphere data, a region between PsaL and PsaD is altered, possibly forming a tighter conformation and contributing to the decreased rates of flavodoxin (Fld) photoreduction in *in vitro* assays (15). This could arise from longer-range modifications to how PsaD and PsaC interact (*via* the PsaD arm that wraps around PsaC), possibly affecting the binding of ferredoxin (Fd) (28) or its interaction with PsaC. Further, adjustments in the relative position or conformation of PsaD may impact interaction with PBS, since PBS has been shown to form contacts with PsaD (29), potentially decreasing energy transfer from PBS to ORR1 PSI. Consistent with this, we have also observed changes in the preference of the CpcL-PBS form for PSI and PSII isolated from the ORR1 strain (30). This may represent a functional mechanism for attenuating light harvesting at PSI to minimize electrons transferred beyond PSI and/or minimizing damage caused by overexcitation of PSI, especially when the protective peripheral electron consumption pathway is absent. Thus, our findings support the hypothesis that changes in the PsaL protein environment serve to adjust PSI photochemistry to balance electron flux under dynamic conditions.

Plasticity in PSI oligomeric form may act as a functional advantage. This is supported by evidence of intact and undamaged terminal iron-sulfur clusters in ORR1 PSI monomers, challenging paradigms that often attribute monomerization to damage. Further, our work illustrates that there are distinct differences between PSI monomers generated *via* genetic manipulation of *psaL* and those arising from physiological conditions (15). Other reports have shown that the absence of PsaL contributes to modifications of PSI photochemistry, such as reduced far-red absorbance (22) or changes in the PSI-associated electron transfer rate (31). Interestingly, in the latter study, the flavodiiron-1 gene, which mediates O_2_ photoreduction activity (*i.e.* ORR1), was seen to be downregulated in the *psaL*-less strain of *S.* 6803 (31). However, whether the downregulation is caused by monomerization or specifically by the loss of the PsaL subunit is not known. Furthermore, the functional relevance of this connection between flavodiiron-1 and PSI monomerization represents an intriguing new area for better understanding the regulation and control of PSI properties. Our work suggests that the loss of electron utilization pathways has a direct impact on the oligomeric and photochemical properties of PSI. In intact PSI monomers, changes in chl and carotenoid pigment properties and abundance, enabled by fine modulations of the PsaL environment, constitute a functional mechanism to respond to altered electron flow by essentially downregulating excitation energy transfer to PSI reaction centers.

Perturbations in the ORR1 PsaL protein environment at the C-terminal region of the subunit, reported as ASN144-ASN157 in WT (19), may enable the shift towards a greater proportion of monomeric PSI in ORR1 (15). In ORR1 PSI monomers, the increased accessibility of PsaI, a subunit which also contributes to the PSI trimeric core (19, 32, 33, 34), may be additionally responsible for the shift towards more monomeric content. While PsaL was retained and not genetically manipulated in our study, others have observed that manipulations of PsaL can have profound effects on both pigment and oligomeric composition. A previous study found that the addition of a His residue to the C-terminus resulted in the loss of two β-carotenes and the equivalent of two chls associated with monomeric PsaL, shifts in the relative positions of PsaI and carotenoids associated with PsaI and PsaM, and decreased trimerization (19).

Further, our findings show changes in the accessibility of PsaL in ORR1 at residues purported to be associated with trimer stability. It has been previously proposed that a Ca^2+^ ion coordinated by PsaL residues PRO70 and ASP73 is involved in trimer stabilization (35, 36). In the crystal structure of *S.* 6803 trimer, this Ca^2+^ ion was coordinated by these residues and was located near PsaL residues of the adjacent monomer, ASP149, ARG153, and the PsaL C-terminus (PHE156, ASN157) (11). Our data showed that four of these residues were in regions with increased accessibility in the ORR1 monomer (PRO70, ASP73, ASP149, ARG153), and two of these residues were in regions with increased accessibility in the WT monomer (PRO70, ASP73). Additionally, the previous study reported that the above C-terminal residues were adjacent to residues in PsaA (ARG466, GLN468, ASP 469) and PsaB (Pro94, His94) (11). Our findings show that these were not detected in our mass spectrometric analysis in the interaction sphere around PsaL in WT monomers or ORR1 trimers and monomers. Our results are consistent with these previous assertions of specific residues that enable the stabilization of trimeric PSI. Combined, our findings show how changes in protein environment at the PSI monomer-monomer interface may enable adjustments in oligomeric form as an adaptive response and may shed light on the assembly/disassembly processes of PSI.

In this context of adjustments in the PsaL protein environment together with decreased photooxidative capacity, the changes in carotenoid abundance and composition may serve to dissipate energy when photon availability and photoexcitation outmatch the electron utilization capability. The increased echinenone and zeaxanthin in ORR1 PSI trimers may alleviate excess energy rather than promoting transfer to P_700_, and possibly PSII. Further supporting this model, we show that zeaxanthin and echinenone were also increased in WT PSI, albeit to a lesser extent than in ORR1 PSI, when cultured under FL conditions, as additional evidence that these carotenoids facilitate the alleviation of excess photon energy. These results are congruent with a previous report of increased levels of these carotenoids in a study of WT *S*. 6803 PSII under increased light intensity (37), and previous studies attributing zeaxanthin and echinenone a role in dissipating excess photon energy (38, 39, 40, 41). Additionally, the decreased β-carotene content observed in ORR1 monomers under FL vs GL could potentially be connected to the two β-carotenes in the PsaL, which correspond to increased accessibility. While these changes cannot be assigned to specific carotenoids in relation to distinct subunits without a complete structural analysis, the observed alterations in pigment composition are consistent with previous reports and could arise due to changes like we observe in the protein environment of PsaL.

The plasticity of the PsaL protein environment functions as a mechanism to modulate PSI oligomeric form, spectral and photochemical properties to preserve functionality and tune electron management at PSI. Additional studies are needed to interrogate specific pigment compositions and interactions with subunits more fully, as well as to comprehensively explore all subunit-subunit interactions particularly in the trimerization core. These investigations will provide further insights into the functional range of flexibility and adjustment of the protein environment of PSI for tuning energy management. Further, our findings show a difference between PSI monomers and trimers in the changes to the PsaL protein environment and interactions with other subunits that occur in response to the loss of the ORR1 pathway in combination with increased light. Collectively, our analyses point to a cumulative effect of protein-protein and protein-cofactor interactions that govern the modulation of PSI oligomeric form. These differences in responses and the expected consequences of these variations further point to the functionality of both monomeric and trimeric PSI, and the contributions of flexible oligomeric form in energy management at PSI. Such flexibility has been observed with other components of the PET chain, such as the two forms of PBS that have different preferences for the PSI and PSII reaction centers (30, 42, 43), as well as the small and long isoforms of Fd:NADP^+^ oxidoreductase (FNR) enzymes (44, 45, 46, 47), and the observation of various supercomplexes (48). These examples facilitate specific energy transformation processes under distinct intracellular redox and/or light conditions. The influence of PSI oligomeric composition and its ability to modulate energy and electron flow in analogous ways is underexplored and the work presented here expands upon this knowledge.

## Conclusion

This work demonstrates that naturally occurring adjustments in the protein environment of PsaL and PSI pigment composition contribute to the control of excitation energy at PSI under dynamic changes in electron demand and photon availability. Further, this points to a greater diversity in the structural and spectral properties among PSI forms than previously realized. This diversity may represent various mechanisms for controlling PSI photochemical response to dynamic conditions of electron demand and photon availability in both trimers and monomers. In the absence of the ORR1 pathway, both modulation of PsaL accessibility and reaction center pigment compositions appear to work cohesively to release excitation pressure, allowing for sustained, albeit lowered, PET activity. Evidence of diversity in PSI oligomeric structures demonstrates a need to define the interactions and energy transfer pathways more fully between subunits and cofactors. While the presence of PsaL is important for dictating the oligomeric state of PSI, our work highlights that more subtle changes in PsaL structure, flexibility, dynamics and interactions also have impacts on PSI oligomeric structure and functional properties. Since each monomer of a trimeric PSI (*i.e.* a protomer) experiences a slightly different environment including unique contacts with nearby residues resulting in distinct interaction networks, salient questions arise regarding the management of excitation energy from the antenna to P_700_ in both the same protomer and across protomers. Furthermore, since a spatially separated monomer is not simply a protomer that is free from a trimer, the monomers investigated here represent a functional form with specific properties that facilitate energy transforming reactions in a manner different from trimeric PSI. Addressing these questions will facilitate an understanding of the PSI-specific and broader mechanisms underlying dynamic responses to fluctuating external stimuli that can support new approaches for utilizing photosynthetic organisms for renewable energy applications. Understanding the many nuances of how energy is controlled at PSI, which have been revealed here, are essential to advancing engineering approaches that rely on efficient conversion of light to drive flux through metabolic pathways, particularly when manipulating electron utilization reactions (1, 2, 3, 4, 5, 6).

## Experimental procedures

### Strains and culture conditions

*Synechocystis* sp. PCC 6803 (*S*. 6803) WT and a strain deficient in Flv1, ORR1, were used for the following experiments, with strains generated as described in (15). Strains were maintained on BG-11 (49) supplemented with 20 mM NaHCO_3_, 20 mM TES (pH 6.8), and appropriate antibiotics (25 μg L^−1^ spectinomycin for ORR1). Cultures were initially grown in a Percival chamber in plates and 250 ml flasks at 23 °C, 50 μmol photons m^-2^s^-1^ white LED light, in air supplemented with 5% CO_2_, with flasks shaken at 120 rpm. Experimental conditions included two light regimes: standard growth conditions using 35 μmol photons m^−2^s^−1^ continuous light (GL), and increased photon flux using cycles of 35 μmol photons m^−2^s^−1^ for 5 min and 500 μmol photons m^−2^s^−1^ for 30 s to produce fluctuating light (FL). Strains were cultured under GL for 36 h, then under GL or FL for 96 h. Cultures were grown in custom round-bottom bioreactor bottles, using a volume of 1250 ml per bottle, in air supplemented with 3% CO_2_ (with bubbling), with stirring, and illumination from the side using white LED lights.

### Isolation of thylakoid membranes

Thylakoid membranes (TMs) were isolated from cells as previously described (15). Cells were harvested by centrifugation at 5000×*g* for 10 min at 4 °C, and the pellet was resuspended in 20 mM HEPES-NaOH buffer, pH 7.5, with 10 mM CaCl_2_, 10 mM MgCl_2_, 10 mM NaCl, and 15% glycerol, and then frozen in liquid N_2_ and stored at −80 °C. Cells were lysed using three passes through a French Press, with the samples on ice. Cell lysate was centrifuged at 5000×*g* for 10 min at 4 °C, and the supernatant collected and ultracentrifuged at 208,000×*g* for 1 h at 4 °C (Type 70 Ti rotor; Beckman Coulter). The pellet containing TMs was resuspended in the same Hepes buffer to 1 mg chl ml^−1^. The suspension was adjusted to include n-dodecyl-β-D-maltoside (DDM) (DDM25, GoldBio) at a final concentration of 1%, and the chl concentration adjusted to 0.4 to 0.5 mg ml^-1^. Samples were incubated with gentle stirring for 2 h at 4 °C in darkness to solubilize TMs. Samples were centrifuged at 12,000×*g* for 20 min at 4 °C to pellet non-solubilized material, and the supernatant was collected. Isolated TMs were frozen in liquid N_2_ and stored at −80 °C.

### Isolation of PSI monomers and trimers

PSI trimers and monomers were isolated from TMs using anion-exchange chromatography. Solubilized TMs were loaded onto a packed DEAE column (17-0709-01, GE Healthcare), with a column volume (CV) of 150 ml, on a chromatography system (AKTA Purifier 10), run with Unicorn software (GE Healthcare). A flow rate of 4 to 5 ml min^−1^ was used for all steps, except a flow rate of 2 ml min^-1^ was used to load samples. The column was equilibrated with 3 CVs of equilibration buffer (20 mM HEPES-NaOH buffer, pH 7.5, with 10 mM CaCl_2_, 10 mM MgCl_2_, 10 mM NaCl, and 0.04% DDM). Solubilized TMs were loaded onto the column using equivalent amounts of chl (2 mg). The column was washed with 2 CVs of equilibration buffer. Two different fractions were eluted (the first containing PSI monomers and PSII, and the second containing PSI trimers) using a linear gradient of 0 mM to 300 mM MgSO_4_. Buffer A was 20 mM HEPES buffer, pH 7.5, containing 10 mM CaCl_2_, 10 mM MgCl_2_, 10 mM NaCl, 0.04% DDM. Buffer B was 20 mM HEPES buffer, pH. 7.5, 10 mM CaCl_2_, 10 mM MgCl_2_, 10 mM NaCl, 0.04% DDM, and 500 mM MgSO_4._ A linear gradient was used to increase the gradient from 0 mM MgSO_4_ to 300 mM MgSO_4_ to elute PSI trimers, and PSI monomers and PSII, followed by a step increase to 500 mM MgSO_4_ to elute remaining proteins from the column, similar to (50). Eluted samples were dialyzed against equilibration buffer for 16 h at 4 °C (132542, Spectrum Labs). Samples were concentrated using centrifugal filtration units (901024, 503096, EMD Millipore), and then frozen in equilibration buffer containing 10% glycerol in liquid N_2_ and stored at −80 °C.

### EPR sample preparation and spectroscopy

Reduced ORR1 monomers and trimers were prepared by exchange into buffer containing 5 mM NaDT, followed by ∼5 to 10 min incubation in the dark on ice. EPR samples were then prepared by loading of ∼200 μl into EPR tubes (Wilmad lab glass, 707-SQ-250 mm) under ambient room light and freezing in liquid N_2_. Samples were kept in the dark until collection. Sample chlorophyll concentrations were determined at 1.1 mg chl ml^-1^ (trimers) or 2.02 mg chl ml^-1^ (monomers). X-band (∼9.38 GHz) CW EPR data were collected on an Elexsys E500 spectrometer that has a super high-Q resonator (Bruker), cryogen-free helium system (ColdEdge Technologies), and MercuryiTC temperature controller (Oxford Instruments). Spectra were collected using a modulation frequency of 100 kHz and modulation amplitude of 10 G. Data were baseline corrected by subtraction of a polynomial function in Igor Pro v.9 (Wavemetrics).

### Mass spectrometry

PSI composition as well as complex stability were investigated on the peptide level. The same amount of PSI (10 μg based on total protein concentration) was incubated with pepsin (2.5 μg activated at pH 2) at room temperature for 5 or 10 min. The digested samples were immediately injected on a reverse phase column (Onyx Monolithic C18, 100 mm × 2 mm; Phenomenex) without any quenching. Peptide mixtures were analyzed on a 1290 ultrahigh pressure series chromatography stack (Agilent Technologies) coupled directly to 1290 Infinity DAD UV/Vis detector (Agilent Technologies) and ESI-TOF mass spectrometer (Micro-TOF, Bruker Daltonics). Peptides were separated at 50 °C using a flow rate of 600 μl/min under the following conditions: 1.0 min, 5% B; 1.0 to 20.0 min, 5 to 30% B; 20.0 to 25.0 min, 30 to 90% B; 25.0 to 27.0 min, 90.0 to 95.0% B; 27.0 to 27.1 min, 95 to 5% B, 27.1 to 30.0 min, 5% B. Solvent A comprised 0.1% formic acid (FA, Sigma) in water (ThermoFisher) and solvent B comprised 0.1% FA in acetonitrile (ThermoFisher). Electrospray conditions were as follows: nebulizer 6.0 bar, drying gas at a flow rate of 6.0 L/min, drying temperature at 200 °C, capillary voltage at 4.5 kV, capillary exit voltage at 150 V, skimmer one set at 60 V, skimmer two set at 22 V, set at hexapole RF 350 V and lens transfer at 87 μsec. Peptide identification was performed using the peptide analysis worksheet for single level MS (PAWs, ProteoMetrics LLC.) with 10 ppm mass measurement error for parent ion. Molecular graphics were created using PyMOL 2.5.0.

### Extraction of pigments

Samples of PSI monomers and trimers isolated using anion-exchange chromatography, in amounts of 0.1 to 0.2 mg chl, were freeze-dried (7670520, Labconco). Pigments were extracted using 80% acetone in water. Samples were then centrifuged at 17,000×*g* for 6 min, and the supernatant collected. In glass tubes, samples were dried under N_2_ gas in darkness and then resuspended in 2 ml HPLC-grade ethanol. Samples were filtered using a 0.2 μm pore size PTFE syringe filter.

### UV-VIS absorbance analysis

Absorbance spectra of samples containing intact PSI were collected at room temperature using a UV-VIS spectrophotometer (DU800, Beckman Coulter), using diluted samples in the buffer used for the isolation of PSI fractions (20 mM HEPES-NaOH buffer, pH 7.5, with 10 mM CaCl_2_, 10 mM MgCl_2_, 10 mM NaCl, and 0.04% DDM). Spectra were collected between 300 and 800 nm and were normalized to the peaks for chl*a* at 676 to 678 nm. Data were collected for biological triplicates and averaged (Figs, 5 and S2).

### HPLC analysis of pigments

Pigment analysis was performed using high-pressure liquid chromatography (HPLC) on a 1200 Series Agilent system with a Synergi Hydro-RP 80A 250 × 4.6 mm column (Phenomenex) (similar to (21)). The column was equilibrated with a mobile phase of acetonitrile:water:triethylamine (9:1:0.01) (Buffer A). Sample injection volumes were 100 μl. Pigments were eluted from the column with a linear gradient from 0 to 100% ethyl acetate (Buffer B) in 25 min, using a constant flow rate of 1 ml min^−1^. Following the elution of each sample, the column was re-equilibrated for 20 min with Buffer A. A MWD monitored multiple wavelengths, and data collected at 454 nm, 460 nm, and 450 nm were used for differentiating and quantifying β-carotene, echinenone, and zeaxanthin respectively. Pigments were identified based on retention time. Standards used were β-carotene (C4582, Sigma Aldrich), echinenone (73341, Sigma Aldrich), and zeaxanthin (144-68-3, Fisher Scientific), each prepared in HPLC-grade ethanol. Concentrations of these carotenoids were calculated using molar extinction coefficients (as reported in (51, 52)). Samples were run as biological triplicates for each strain, condition, and oligomeric form, and values were calculated as molecules of pigment per 100 molecules of chl*a*. Data were collected for biological triplicates. Data are shown as the average of biological triplicates, except for ORR1 FL trimer for echinenone and zeaxanthin, and ORR1 Gl trimer for β-carotene, which have two biological replicates to exclude outlier values, with error bars showing standard deviation (Figs. 10, S4, and Table S6).

## Data availability

All data are contained within the manuscript and supporting information.

## Supporting information

This article contains supporting information (11, 53).

## Conflict of interest

The authors declare that they have no conflicts of interest with the contents of this article.

## Supporting information

Supporting Information

PsaL-5A-interaction-sphere
