# Supplementary material for: Modulation of cyanobacterial Photosystem I protein environment and spectral capacity in response to changes in electron flow pathways and photon flux
Source: J Biol Chem. 2025 May 14;301(7):110233. doi: 10.1016/j.jbc.2025.110233 (PMC12212252; doi:10.1016/j.jbc.2025.110233)
Supplement: Supporting Information [file mmc1.docx]

Supporting Information for:

**Modulation of cyanobacterial Photosystem I protein environment and spectral capacity in response to changes in electron flow pathways and photon flux**

Sharon L. Smolinski, Monika Tokmina-Lukaszewska, Junia M. Holland, Zhanjun Guo, Effie Kisgeropoulos, Brian Bothner, Paul W. King and Carolyn E. Lubner

Corresponding Author: Carolyn E. Lubner

This PDF file includes:

Figures S1 - S4

Tables S1 – S6

Mass spectrometric data workflow

References

Table of Contents

Figure S1………………………………………………………………………………………………………………………………………............2

Figure S2………………………………………………………………………………………………………………………………………............3

Figure S3………………………………………………………………………………………………………………………………………............4

Figure S4………………………………………………………………………………………………………………………………………............5

Table S1………………………….…………………………………………………….…………………………………………………………………6

Table S2.………………………………………………………………………………………………………………………………………………….7

Table S3…………………………………………………………………………………………………………………………………………………..8

Table S4…………………………………………………………………………………………………………………………………………………..9

Table S5…………………………………………………………………………………………………………………………….…………………..10

Table S6…………………………………………………………………………………………………………………………………………………11

References………………………………………………………………………………………………………………………………………….…12

**Supplementary Figures**

**
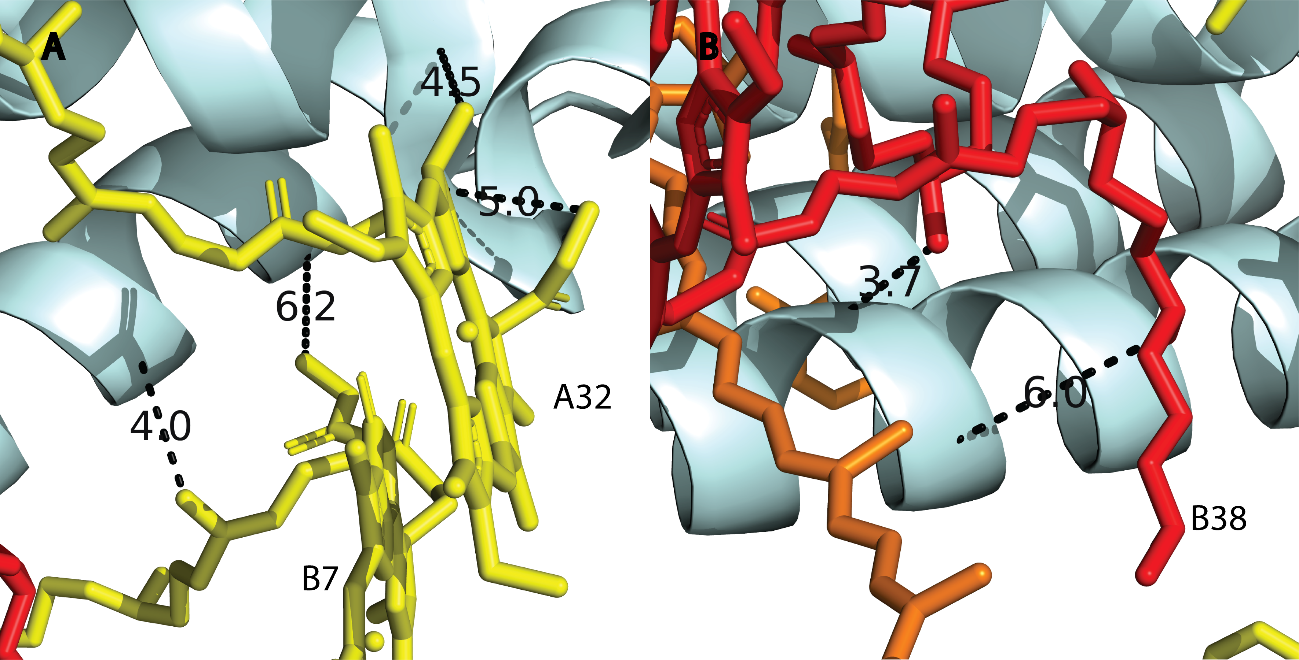
**

**Figure S1**. **Proximity of select chls to PsaL**. Proximity of red chl pairs A) A32/B7 (yellow) and B) B37/B38 (red) to PsaL (cyan), based on the reported structure for *S*. 6803 WT PsaL from PDB 5OY0 (1), with distances in Å, and β-carotene in orange.


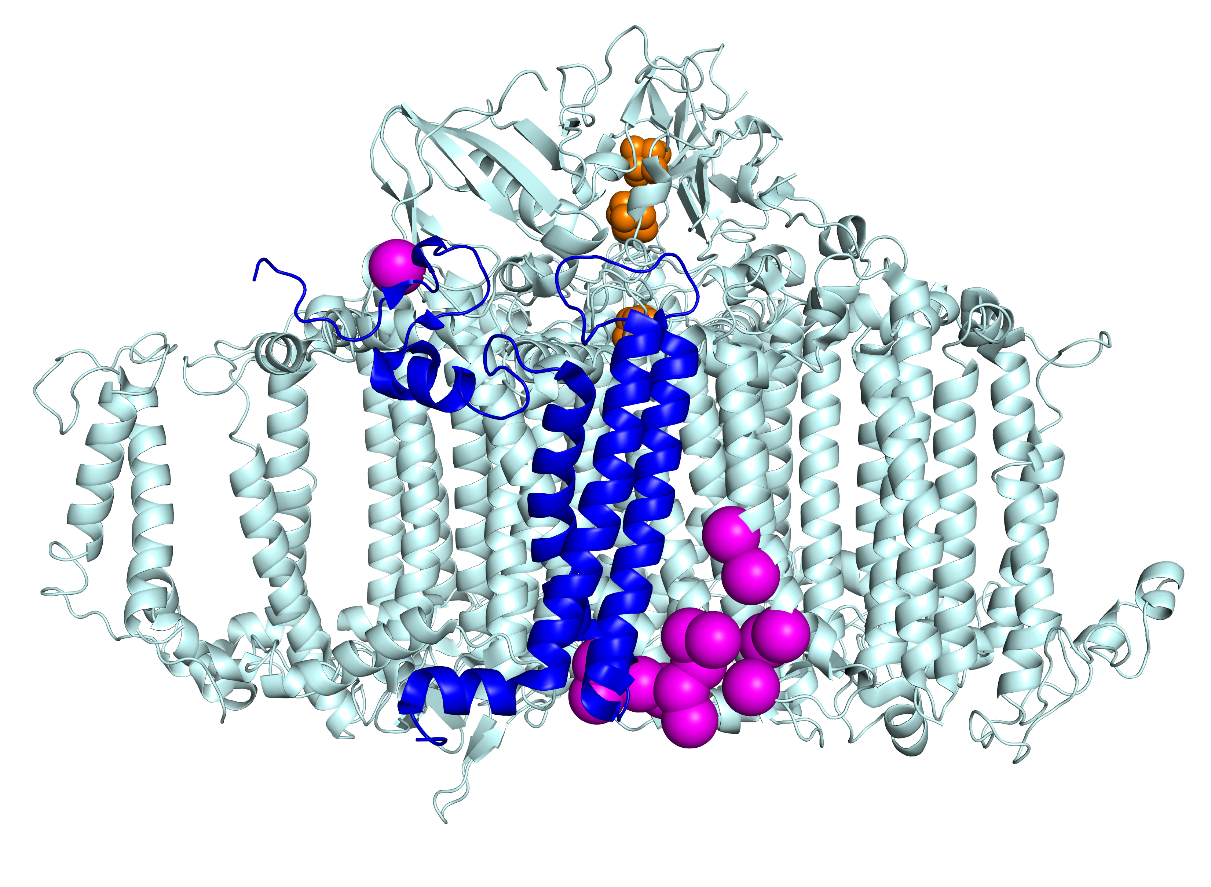


**Fig S2.** **The detection of residues** **within 5 Å of PsaL in WT PSI monomers compared to WT PSI trimers.** Residues within the PsaL interaction sphere that were not detected in WT PSI monomer compared but were detected in WT PSI trimers are shown in magenta, based on mass spectrometric analysis of isolated PSI following proteolytic digestion. PsaL is shown in blue, and iron-sulfur clusters are shown in yellow. The mass spectrometric data were mapped onto a monomeric unit based on the structure of *S*. 6803 WT PsaL from PDB 5OY0 (1). PSI is shown with the stromal side on top.

**
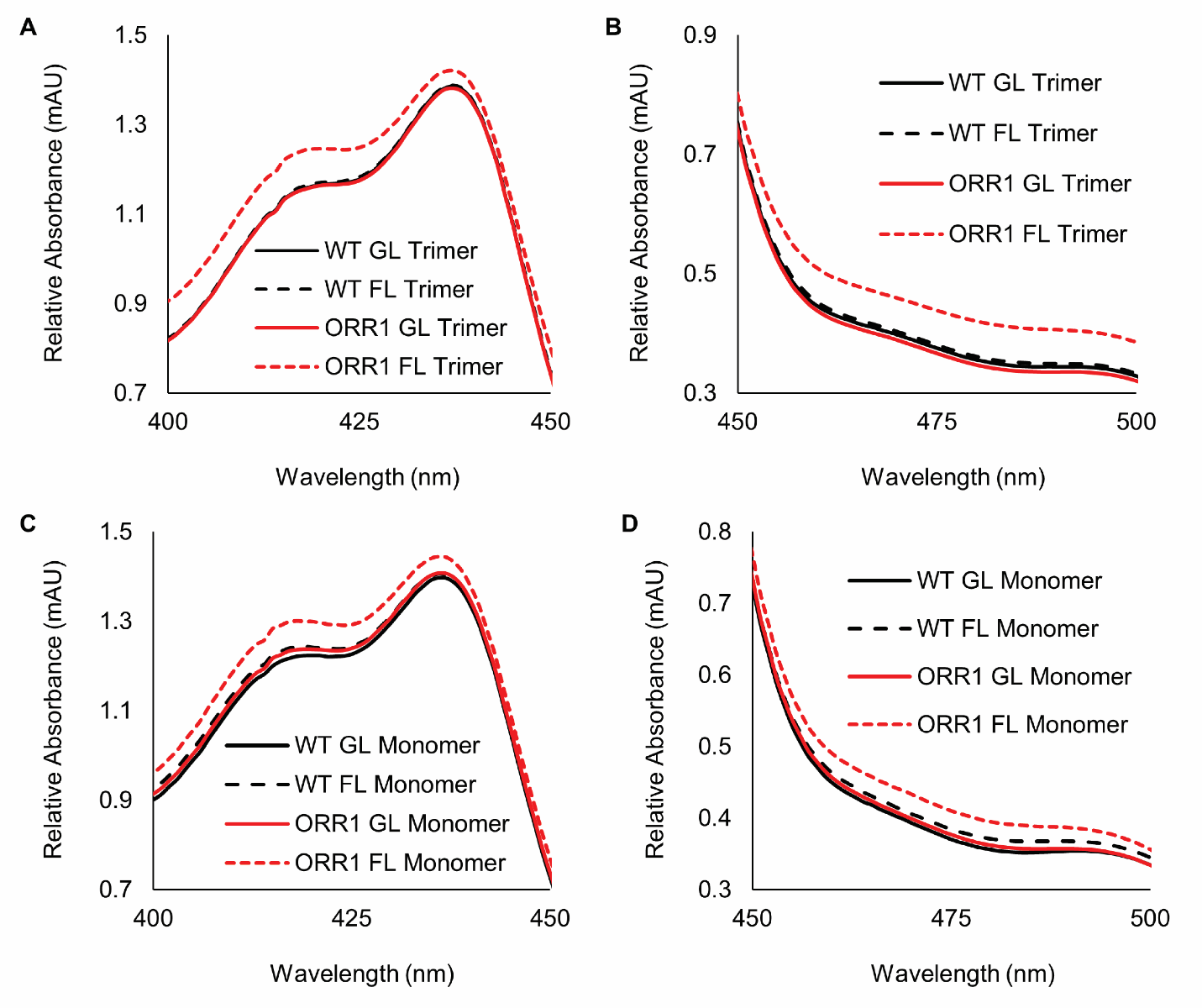
**

**Figure S3**. **UV-VIS spectra of isolated PSI showing pigment characteristics**. UV-VIS spectra showing relative absorbance over 400-500 nm of intact and isolated PSI trimers, and PSI monomers (also containing PSII) from WT and ORR1 cells grown under continuous moderate light (GL) or increased and fluctuating light (FL). Absorbance by PSI trimers overs A) 400-450 nm, and B) 450-500nm. Absorbance by PSI monomers over C) 400-450nm, and D) 450-500 nm. ORR1 PSI trimers, and ORR1 PSI monomers (with PSII) show increased absorbance 400 to 440 nm and 460 nm to 500 nm. Data were normalized to the peak for chl *a*, at 676 nm to 678 nm. Data are the average of biological triplicates.

**
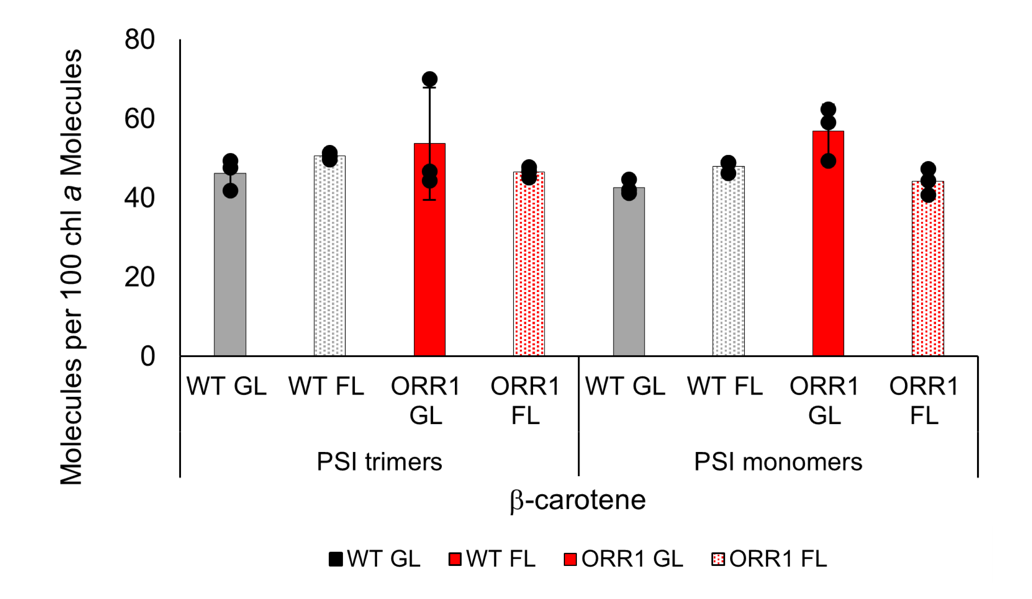
**

**Figure S4.** The abundance of β-carotene in pigments extracted from samples containing PSI. Samples of PSI trimers and monomers were isolated from WT (black) and ORR1 (red) cells grown under GL (solid) and FL (pattern) conditions. Data are based on absorbance at 454 nm and quantified as the number of molecules per 100 chl*a* molecules. Data are the average of biological triplicates, with the exception of ORR1 GL trimer for β-carotene which have two biological replicates to exclude outlier values, with error bars showing standard deviation.

**Supplementary Tables**

**Table S1. PSI ligands depicted in Figure 1B.**  Ligands shown are based on the structure for WT *S*. 6803 PSI Trimer PDB 5Oy0 (1).

| Subunit | Type | Ligand | Label in Figure |
| --- | --- | --- | --- |
| PsaA | Chlorophyll | CLA 1011 |  |
|  | Chlorophyll | CLA 1012 |  |
|  | Chlorophyll | CLA 1013 |  |
|  | Chlorophyll | CLA 1132 | A32 |
|  | Fe-S cluster | SF4 3001 | F_X_ |
|  | Phylloquinone | PQN 2001 |  |
| PsaB | Chlorophyll | CLA 1021 |  |
|  | Chlorophyll | CLA 1022 |  |
|  | Chlorophyll | CLA 1023 |  |
|  | Chlorophyll | CLA 1207 | B7 |
|  | Chlorophyll | CLA 1237 | B37 |
|  | Chlorophyll | CLA 1283 | B38 |
|  | Phylloquinone | PQN 2002 |  |
| PsaC | Fe-S cluster | SF4 3002 | F_A_ |
|  | Fe-S cluster | SF4 3003 | F_B_ |
| PsaL | β-carotene | BCR 4019 |  |
|  | β-carotene | BCR 4022 |  |
|  | Chlorophyll | CLA 1501 |  |
|  | Chlorophyll | CLA 1502 |  |
|  | Chlorophyll | CLA 1503 |  |

**Table S2. Predicted cleavage sites of WT *S*. 6803 PsaL by pepsin**. Data from Expasy (2).

| Proteolysis conditions (pH) | Number of cleavage sites | Positions of cleavage sites |
| --- | --- | --- |
| > 2.0 | 61 | 10, 11, 15, 19, 20, 28, 29, 32, 37, 40, 48, 50, 58, 60, 61, 64, 66, 67, 70, 75, 76, 77, 78, 81, 82, 85, 86, 87, 88, 89, 90, 95, 96, 98, 99, 100, 101, 103, 104, 113, 114, 119, 120, 122, 123, 126, 127, 128, 135, 136, 138, 139, 140, 141, 142, 144, 145, 151, 152, 154, 156 |

**Table S3. The detection of PSI residues within 5 Å of PsaL in ORR1 PSI monomers compared to WT PSI monomers.** Seventeen residues were not detected in ORR1 PSI monomers, but were detected in WT monomers. Six residues were detected in ORR1 PSI monomers but not detected in WT monomers. Based on mass spectrometric analysis of PSI isolated from cells grown under increased and fluctuating light conditions (FL), and following proteolytic digestion of the isolated protein.

| Subunit | Residues not detected in ORR1 PSI monomers, but detected in WT PSI monomers  (for one monomeric unit) | Residues detected in ORR1 PSI monomers, but not detected in WT PSI monomers  (for one monomeric unit) |
| --- | --- | --- |
| A | HIS 432  ARG 560 |  |
| B | TRP 677  ARG 681 | TRP 92 |
| D | PRO 8  PRO 9  PHE 11  GLY 13  SER 14  THR 15  GLY 16  GLY 17  LEU 18  LEU 19  MET 42  LEU 57  TYR 58 |  |
| I |  | MET 1  ASP 2  TRP 12  PRO 16  TRP 20 |

**Table S4. The detection of PSI residues within 5 Å of PsaL in ORR1 PSI trimers compared to WT PSI trimers.** Nine PSI residues were not detected in ORR1 PSI trimers, but which were detected in WT trimers. Based on mass spectrometric analysis of PSI isolated from cells grown under increased and fluctuating light conditions (FL), and following proteolytic digestion of the isolated protein.

| Subunit | Residues not detected in ORR1 PSI trimers, but detected in WT PSI trimers  (for one monomeric unit) |
| --- | --- |
| A | GLY 465  ARG 466  GLN 468  ASP 469 |
| B | PRO 94  HIS 95  PHE 96  GLY 97  GLU 98 |

**Table S5. The detection of PSI residues within 5 Å of PsaL in WT PSI monomers compared to WT PSI trimers.** Fifteen PSI residues within 5 Å of PsaL were not detected in WT PSI monomers, but were detected in WT trimers. Based on mass spectrometric analysis of PSI isolated from cells grown under increased and fluctuating light conditions (FL), and following proteolytic digestion of the isolated protein.

| Subunit | Residues not detected in WT PSI monomers, but detected in WT PSI trimers  (for one monomeric unit) |
| --- | --- |
| A | GLY 465  ARG 466  GLN 468  ASP 469 |
| B | TRP 92  PRO 94  HIS 95  PHE 96  GLY 97  GLU 98 |
| D | GLY 12 |
| I | MET 1  TRP 12  PRO 16  TRP 20 |

**Table S6. The abundance of select carotenoids in samples containing PSI monomers or trimers**. pigments extracted from samples containing PSI trimers and monomers isolated from WT and ORR1 cells grown under GL and FL. Data are quantified as the number of molecules per 100 chl *a* molecules, and shown as a percentage compared to WT GL. Data are the average of biological triplicates, with the exception of: zeaxanthin ORR1 FL trimer and β-carotene ORR1 GL trimer which both have two biological sample, ± standard deviation.

|  | WT GL | ORR1 GL | WT FL | ORR1 FL |
| --- | --- | --- | --- | --- |
| Echinenone | | | | |
| Trimer | 5.8 ± 0.1 (100%) | 5.8 ± 0.8 (100%) | 7.3 ± 0.5 (126%) | 8.4 ± 0.9 (145%) |
| Monomer | 4.6 ± 0.1 (100%) | 4.9 ± 0.6 (107%) | 5.6 ± 0.2 (122%) | 8.2 ± 1.3 (178%) |
| Zeaxanthin | | | | |
| Trimer | 3.7 ± 0.4 (100%) | 3.1 ± 0.6 (84%) | 3.9 ± 0.2 (105%) | 5.9 ± 0.9 (159%) |
| Monomer | 3.1 ± 0.5 (100%) | 3.6 ± 1.1 (116%) | 4.2 ± 1.2 (135%) | 6.3 ± 0.4 (203%) |
| β-carotene | | | | |
| Trimer | 46.2 ± 4.1 (100%) | 45.5 ± 1.8 (98%) | 50.5 ± 0.9 (109%) | 46.5 ± 1.3 (101%) |
| Monomer | 43.9 ± 1.8 (100%) | 58.7 ± 6.9 (134%) | 49.5 ± 1.5 (113%) | 45.5 ± 3.4 (104%) |

Mass spectrometric data workflow

The workbook “PsaL-5A-interaction-sphere.xlsx” contains the data from the PsaL interaction sphere experiment including the workflow for processing and analysing the detected/undetected peptides data. Data includes the generation of the interaction reference list for each of the three PsaL subunits in trimeric PSI, pepsin digestion patterns for each sample, and pairwise comparisons of the undetected regions of the PsaL interaction sphere to reveal residues that were detected in one strain and form but not in another.

**References**

1. Malavath, T., Caspy, I., Netzer-El, S. Y., Klaiman, D., and Nelson, N. (2018) Structure and function of wild-type and subunit-depleted photosystem I in Synechocystis. *Biochim. Biophys. Acta - Bioenerg.* **1859**, 645-654.

2. Expasy. (n.d.) PeptideCutter.
